# Is Cirrhotic Cardiomyopathy Related to Cirrhosis Severity?

**DOI:** 10.5041/RMMJ.10488

**Published:** 2023-01-29

**Authors:** Subhash Chandra Dash, Beeravelli Rajesh, Suresh Kumar Behera, Naba Kishore Sundaray, Praveen Patil

**Affiliations:** 1 Department of General Medicine, Institute of Medical Sciences & SUM Hospital, Bhubaneswar, India; 2 Department of General Medicine, Chalmeda Ananda Rao Institute of Medical Sciences, Telangana, India; 3 Department of Cardiology, Institute of Medical Sciences & SUM Hospital, Bhubaneswar, India; 4 Department of Neurology, Jawaharlal Nehru Medical College, Karnataka, India

**Keywords:** Alcoholic cirrhosis, cardiac dysfunctions, decompensated cirrhosis, diastolic dysfunction, non-alcoholic, systolic dysfunction

## Abstract

**Objective:**

Cirrhotic cardiomyopathy (CCM) is associated with increased morbidity and mortality in patients with liver cirrhosis. Yet, it remains an under-diagnosed entity. Further, its relation to the severity of cirrhosis is contradictory. We conducted this study on an Indian population to determine the cardiac dysfunctions in cirrhosis of the liver and correlations with etiologies and cirrhosis severity.

**Methods:**

This study enrolled patients with diagnosed liver cirrhosis without any cardiac disease or conditions affecting cardiac function. All participants were evaluated clinically, electrocardiographically, and echocardiographically. Cirrhosis severity was assessed by scores from the Model for End-stage Liver Disease (MELD) and Child–Turcotte–Pugh (CTP) tests. Cirrhotic cardiomyopathy was defined as diastolic dysfunction and/or systolic dysfunction with QT prolongation.

**Results:**

Ninety-six patients were evaluated, and CTP-A stage of cirrhosis was found in 23 (24%), CTP-B in 42 (43.8%), and CTP-C in 31 (32.3%) cases. Systolic dysfunction was most frequent (*P*=0.014), and left ventricular ejection fraction was significantly reduced (*P*=0.001) in CTP-C stage of cirrhosis. Cirrhotic cardiomyopathy was found in 39.6% (*n*=38) of patients; CCM patients had significantly higher CTP scores (9.6±2.6 versus 8.3±2.3, *P*=0.012) as well as MELD scores (19.72±4.9 versus 17.41±4.1, *P*=0.015) in comparison to patients without CCM.

**Conclusion:**

Cirrhotic cardiomyopathy has a positive relationship with the severity of cirrhosis. Systolic function declines with the severity of cirrhosis, and overt systolic dysfunction can be present, particularly in the advanced stage of cirrhosis of the liver.

## INTRODUCTION

Cirrhosis is one of the eventual complications of any chronic liver disease. It is characterized by hepatic necrosis, fibrosis, and regenerative nodules, which lead to circulatory dysfunction and consequent cardiac dysfunctions. Cardiac dysfunctions like high cardiac output, increased circulatory volume, and decreased systemic vascular resistance were first described several decades ago in alcoholic cirrhosis.^1^ Evidence from subsequent experimental and clinical studies determined that these cardiac abnormalities are not due to the toxicity of alcohol but to cirrhosis itself. Impaired cardiac contractility and electrophysiologic abnormalities observed in these studies led to the emergence of the concept of cirrhotic cardiomyopathy (CCM).^1^,^2^

In the initial stage, hyperdynamic circulation develops as a compensatory measure to splanchnic vasodilatation. When cirrhosis progresses, liver dysfunction and portal hypertension cause further vasodilatation, resulting in ineffective blood volume, which triggers the sympathetic nervous system and renin-angiotensin-aldosterone system. These changes aggravate the hyperdynamic circulation and cardiac strain, leading to functional changes in the heart.^1^,^2^ Further studies revealed the pathophysiology as more complex. Impairment of beta-adrenergic receptors, nitric oxide, endocannabinoids, alteration in ion channels function in the myocardium, abnormal myofilaments, inflammatory cytokines, and alteration in lipid metabolism all play a role in CCM pathogenesis.^1^–^4^

In the absence of any concomitant cardiac disease, CCM is characterized by impaired cardiac contractility responsiveness, altered diastolic relaxation, and electrophysiologic abnormalities.^1^ Echocardiography showing diastolic and/or systolic dysfunction is essential for diagnosis of CCM. Diagnostic criteria include evidence of systolic dysfunction (blunted response to stress and decreased resting left ventricular ejection fraction [LVEF]), evidence of diastolic dysfunction (DD) (ratio of early to late atrial flow velocities of less than one, prolonged deceleration time [DT], prolonged isovolumetric relaxation, decreased mitral annular velocity assessed with tissue Doppler imaging), and the presence of various supportive criteria (prolonged QT interval, enlarged left atrium, and increased brain natriuretic peptide and troponin).^1^,^2^ In most patients, systolic dysfunction is subclinical due to reduced afterload but unmasked by physical or pharmacologic stress. Because of this paucisymptomatic manifestation, the exact prevalence of CCM is difficult to determine. However, it may be prevalent in up to 50% of patients with cirrhosis.^4^,^5^ Although much work has been done and reported over the last decade, CCM remains an underdiagnosed entity^4^–^6^ with minimal data on the Indian population.^7^–^10^ Further, the association of CCM with the severity of cirrhosis is contradictory. Some studies observed a positive relationship, while others negated it.^7^,^10^–^12^ This study was conducted to determine the cardiac dysfunctions in cirrhosis of the liver and correlations with etiologies and cirrhosis severity in an Indian population.

## METHODS

A cross-sectional analytical study was carried out in the Institute of Medical Sciences & SUM Hospital, an academic tertiary-care center in Bhubaneswar, India. Consecutive cases of known cirrhosis of the liver admitted to the Department of Medicine and Gastroenterology during May 2018 through October 2019 were included in the study. Cases were diagnosed based on clinical, biochemical, imaging, or histological parameters. Patients aged <18 years and patients with diabetes, hypertension, thyroid disorders, cardiac diseases (coronary artery disease, valvular heart disease, ischemic heart disease, and structural heart disease), chronic pulmonary diseases, renal failure, malignancy, immunodeficiency state, recent bleeding (<3 months), and anemia (hemoglobin of <10 g/dL) were excluded from the study. The study protocol was approved by the institutional ethics committee (ref. DMR/IMS.SH/SOA/180029) and conformed to the ethical guidelines of the Indian Council of Medical Research (ICMR). Written informed consent was obtained from all participants.

### Data Acquisition

Perusal of old medical documents, detailed history-taking, and thorough clinical examination were carried out for all participants. Data regarding age, sex, body mass index, details of alcohol use, etiology of cirrhosis, presence of ascites, gastroesophageal varices, hepatic encephalopathy, heart rate, and systolic–diastolic blood pressure were recorded. Laboratory investigations included complete hemogram, liver biochemistry, blood sugars, HbA1c, renal function test, serum electrolytes, and screening for hepatitis B and C as well as HIV. Cirrhosis severity was assessed by Model for End-stage Liver Disease (MELD) scores and Child–Turcotte–Pugh (CTP) scores and graded as CTP stages A, B, and C. Electrolyte imbalances, if any, were corrected, and diuretics and beta-blockers were withdrawn 48 hours before the electrocardiography and echocardiographic examinations. A 12-lead ECG was recorded in the supine position. Corrected QT (QTc) interval was calculated using Bazett’s formula (QTmax/√RR), and a QTc interval of >440 ms was defined as a prolonged interval. We performed two-dimensional M-mode, pulsed-wave Doppler, and tissue Doppler imaging using a Vivid E9 echocardiography machine (GE Health Care, Chicago, IL, USA) to assess systolic and diastolic functions. Regional wall motion abnormalities and all heart valves were thoroughly observed. Left ventricular diameter and thickness were measured by M-mode. Left ventricular end-diastolic and end-systolic volumes were measured, and left ventricular ejection fraction (LVEF) was estimated using the modified Simpson method. Diastolic function was assessed by measuring peak early filling (E-wave) velocity, late atrial filling (A-wave) velocity, DT, and isovolumetric relaxation time (IVRT) with pulsed-wave Doppler and early diastolic mitral annular velocity (e′) with tissue Doppler examination.

### Operational Definitions

A LVEF of <50% was considered systolic dysfunction. Diastolic dysfunction was defined as per the American Society of Echocardiography guidelines.^13^ Severity of diastolic dysfunction was assessed as grade I with E/A ratio <0.8, DT >200 ms, E/e′<9, and IVRT ≥100 ms; grade II with E/A 0.8–2, DT 160–200 ms, E/e′ 9–12 and IVRT 60–100 ms; and grade III with E/A ≥2, DT <160 ms, E/e′ >12, and IVRT ≤60 ms. The presence of DD and/or systolic dysfunction with QT prolongation was defined as CCM.

### Data Analysis

All the data were analyzed by SPSS version 20.0 (IBM Corp., Armonk, NY, USA). After the normality test, results were presented as frequencies and percentages for categorical data, and as means with standard deviations for continuous data. We compared the categorical variables between patients with CCM and without CCM, alcoholic and non-alcoholic cirrhosis, and stages of liver cirrhosis, using chi-square and Fisher exact tests as applicable. Independent *t*-test and ANOVA test were used to assess the differences between continuous variables among two and three groups, respectively. A two-tailed *P* value of <0.05 denotes a statistically significant difference.

## RESULTS

A total of 96 patients were enrolled in the study. The demographic characteristics and baseline parameters of the study participants are summarized in Table 1. The mean age of the participants was 47.15± 10.6 years, and 36.5% were female. The most common etiology of cirrhosis was alcohol (51.0%, *n*=49), followed by unknown etiology (28.1%, *n*=27), and viral hepatitis (20.8%, *n*=20). Compensated cirrhosis (CTP stage A) was present in 24.0% of patients, and CTP stages B and C were 43.8% and 32.3%, respectively.

**Table 1:** Baseline Parameters of Study Participants (*n*=96).

Parameter	*n* (%)	Mean±SD
Female sex	35 (36.5)	

Age (years)		47.15±10.6

BMI (kg/m^2^)		23.61±3.6

Bilirubin (mg/dL)		2.94±1.18

AST (IU/L)		108.63±23.5

ALT (IU/L)		73.89±23.3

Alkaline phosphatase (IU/L)		159.57±27.1

Albumin (g/dL)		2.71±0.5

INR		1.82±0.5

Creatinine (mg/dL)		1.22±0.2

MELD score		18.32±4.5

CTP score		8.83±2.5

CTP stage		
A	23 (24.0)	
B	42 (43.8)	
C	31 (32.3)	

Etiology		
Alcoholic	49 (51.0)	
HBV	17 (17.7)	
HCV	3 (3.1)	
Unknown	27 (28.1)	

BMI, body mass index; CTP, Child–Turcotte–Pugh; HBV, hepatitis B virus; HCV, hepatitis C virus; INR, international normalized ratio; MELD, Model for End-stage Liver Disease; SD, standard deviation.

Systolic dysfunction was found in 25% (*n*=24) and DD in 45.9% (*n*=44) of patients (grade I 32.3%, grade II 9.4%, and grade III 4.2%). Left ventricular hypertrophy was found in 43.8% (*n*=42) patients. A QT prolongation was found in 61.5% (*n*=59) of patients. Systolic dysfunction, DD, and prolonged QT were found in 9.3% (*n*=9) of cases. Systolic dysfunction or DD with prolonged QT was found in 30.2% (*n*=29) cases. Thus, CCM was found in 39.6% (*n*=38) of patients. Other cardiac parameters are described in Table 2.

**Table 2:** Cardiac Parameters in Study Participants (*n*=96).

Parameter	*n* (%)	Mean±SD
Heart rate (beats/min)		81.65±10.5

Systolic BP (mmHg)		110.96±8.9

Diastolic BP (mmHg)		69±8.8

QTc interval (ms)		428.9±26.4

QTc prolongation	59 (61.5)	

Diastolic dysfunction		
Grade I	31 (32.3)	
Grade II	9 (9.4)	
Grade III	4 (4.2)	

E/A ratio		1.02±0.3

IVRT (ms)		82.66±19.4

DT (ms)		192.42±37.4

E/e′ ratio		7.25±2.0

Systolic dysfunction	24 (25)	

LVEF		55.96±9.2

LVH	42 (43.8)	

CCM	38 (39.6)	

A, late atrial filling velocity; BP, blood pressure; CCM, cirrhotic cardiomyopathy; DT, deceleration time; E, early filling velocity; e′, early diastolic mitral annular velocity; IVRT, isovolumetric relaxation time; LVEF, left ventricular ejection fraction; LVH, left ventricular hypertrophy.

A comparison of clinical and cardiac parameters among the patients in various CTP stages of cirrhosis is shown in Table 3. Systolic dysfunction was most frequent (41.9%, *P*=0.014), and LVEF was significantly reduced (*P*=0.001) in the CTP-C stage of cirrhosis. Also, the QTc interval was progressively prolonged with cirrhosis severity (416.8±25.4 versus 429.5±25.6 versus 437.1±25.5 ms, *P*=0.018). However, DD was not significantly associated with cirrhosis severity (*P*=0.98). Cirrhotic cardiomyopathy was most prevalent in the CTP-C group, reaching statistical significance (54.8%, *P*=0.047). Further, patients with systolic dysfunction had a higher MELD score (21.78±4.3 versus 17.17±4.0, *P*<0.001), and the QTc interval was positively correlated with the MELD score (*r*=0.28, *P*=0.005) (data not shown). Cardiac parameters, CTP classes, and MELD scores were also compared between patients with alcoholic and non-alcoholic cirrhosis, but no significant differences were found (Table 4).

**Table 3:** Comparison of Clinical and Cardiac Parameters with Severity of Cirrhosis.

Parameters	Cirrhosis Stage			χ^2^ / *F* Value	*P* Value

	CTP-A (*n*=23)	CTP-B (*n*=42)	CTP-C (*n*=31)		
Age (years)	47.22±8.9	46.50±12.7	47.97±8.7	0.168	0.846

Female sex	34.7% (8)	42.8% (18)	29.0% (9)	1.508	0.470

BMI (kg/m^2^)	23.63±2.9	23.17±3.4	24.20±4.3	0.704	0.497

Etiology					
Alcoholic	47.8% (11)	47.6% (20)	58.0% (18)	0.904	0.636
Non-alcoholic	52.1% (12)	52.3% (22)	41.9% (13)		

QTc interval (ms)	416.83±25.4	429.48±25.6	437.1±25.5	4.169	0.018*

LVH	30.4% (7)	40.4% (17)	58.0% (18)	4.421	0.110

LVEF	57.0±10.1	58.98±7.5	51.10±8.8	7.585	0.001*

Systolic dysfunction	26.0% (6)	11.9% (5)	41.9% (13)	8.598	0.014*

E/A ratio	0.97±0.3	1.03±0.4	1.05±0.4	0.341	0.731

IVRT (ms)	81.09±19.0	82.50±21.5	84.03±17.2	0.151	0.860

DT (ms)	190.96±33.7	193.64±41.1	191.84±35.8	0.043	0.958

E/e′ ratio	7.15±1.8	7.18±2.0	7.40±2.2	0.137	0.872

Diastolic dysfunction	47.8% (11)	45.2% (19)	45.1% (14)	0.048	0.976

CCM	21.7% (5)	38.0% (16)	54.8% (17)	6.118	0.047*

* Significant; χ^2^, chi-square test; ANOVA test.

A, late atrial filling velocity; CCM, cirrhotic cardiomyopathy; DT, deceleration time; E, early filling velocity; e′, early diastolic mitral annular velocity; *F*, ANOVA test; IVRT, isovolumetric relaxation time; LVEF, left ventricular ejection fraction; LVH, left ventricular hypertrophy.

**Table 4:** Comparison of Cardiac Parameters Between Alcoholic and Non-alcoholic Cirrhosis.

Parameters	Alcoholic Cirrhosis (*n*=49)	Non-alcoholic Cirrhosis (*n*=47)	χ^2^ / *t* value	*P* Value
Heart rate (beat/min)	83.18±12.0	80.04±8.5	−1.470	0.145

Systolic BP (mmHg)	110.94±8.9	110.98±9.1	0.022	0.983

Diastolic BP (mmHg)	70.12±8.4	67.83±9.1	−1.276	0.205

QT prolongation	67.3% (33)	55.3% (26)	1.465	0.226

QTc interval (ms)	432.76±25	424.89±27	−1.467	0.146

Systolic dysfunction	30.6% (15)	19.1% (9)	1.681	0.195

LVEF	56.71±10.2	55.17±8.0	−0.816	0.417

E/A ratio	1.04±0.3	0.99±0.4	−0.622	0.535

IVRT (ms)	79.59±19.6	85.85±18.9	1.588	0.116

DT (ms)	186.55±39.8	198.53±34.2	1.579	0.118

E/e′ ratio	7.07±2.0	7.43±2.0	0.855	0.395

Diastolic dysfunction	40.8% (20)	51.0% (24)	1.015	0.314

CCM	38.7% (19)	40.4% (19)	0.027	0.869

CTP stage				
A	22.4% (11)	25.5% (12)	0.904	0.636
B	40.8% (20)	46.8% (22)		
C	36.7% (18)	27.6% (13)		

MELD score	18.78±4.5	17.85±4.5	−0.988	0.326

χ^2^, chi-square test; *t*, independent *t*-test.

A, late atrial filling velocity; CCM, cirrhotic cardiomyopathy; DT, deceleration time; E, early filling velocity; e′, early diastolic mitral annular velocity; IVRT, isovolumetric relaxation time; LVEF, left ventricular ejection fraction.

Demographic characteristics and etiologies of cirrhosis were not significantly different between patients with and without CCM. But patients with CCM had significantly higher CTP scores (9.6±2.6 versus 8.3±2.3, *P*=0.012) as well as MELD scores (19.72±4.9 versus 17.41±4.1, *P=*0.015) in comparison to patients without CCM (Table 5).

**Table 5:** Demographic and Clinical Characteristics in Patients With and Without Cirrhotic Cardiomyopathy.

Characteristic	Cirrhotic Cardiomyopathy (*n*=38)	No Cirrhotic Cardiomyopathy (*n*=58)	χ^2^ / *t* Value	*P* Value
Age (years)	48.47±10.4	46.28±10.7	0.991	0.324

Female sex	34.2% (13)	37.9% (22)	0.137	0.711

BMI (kg/m^2^)	22.85±3.1	24.11±3.9	−1.659	0.100

Etiology				
Alcoholic	49.0% (19)	53.6% (30)	3.320	0.345
Viral	21.8% (9)	19.5% (11)		
Unknown	29.0% (10)	26.8% (17)		

Bilirubin (mg/dL)	3.0±0.9	2.8±1.3	0.625	0.533

AST (IU/L)	110.30±22.4	107.53±24.3	0.562	0.575

ALT (IU/L)	76.52±19.0	72.17±25.8	0.891	0.375

Albumin (g/dL)	2.6±0.4	2.7±0.5	−1.387	0.169

INR	1.96±0.5	1.72±0.4	2.341	0.021

Creatinine (mg/dL)	1.27±0.2	1.18±0.2	1.934	0.056

CTP score	9.63±2.6	8.31±2.3	2.568	0.012*

MELD Score	19.72±4.9	17.41±4.1	2.469	0.015*

* Significant; χ^2^, chi-square test; *t*, independent *t*-test.

ALT, alanine transaminase; AST, aspartate aminotransferase; BMI, body mass index; CTP, Child–Turcotte–Pugh; INR, international normalized ratio; MELD, Model for End-stage Liver Disease.

## DISCUSSION

The present study represented patients with all stages of cirrhosis (CTP-A in 24.0%, CTP-B in 43.8%, and CTP-C in 32.3%). To date, there is no universal consensus on definitive diagnostic criteria for CCM. Like most previous studies, our study adopted the criteria proposed by the World Congress of Gastroenterology in Montreal.^4^ Our study observed CCM in 39.6% of patients. It was not associated with etiologies of cirrhosis or the demographic characteristics of the patients. However, CCM was significantly more frequent in the advanced stage of cirrhosis (CTP-C) and associated with higher MELD scores as well (*P*<0.05).

Systolic function is most commonly assessed based on LVEF. Unlike other studies on the Indian population, the present study observed systolic dysfunction in 25% of patients.^7^,^10^ It is established that systolic dysfunction is latent in most cases of cirrhosis. Exposure to stressors such as exercise or vasoactive drugs demonstrates a lower increase in LVEF than in normal subjects.^1^ Conversely, several studies found overt systolic dysfunction at rest as well. Hammami et al. observed 17.5% of patients having systolic dysfunction at rest.^14^ Kazankov et al.’s study also observed substantial systolic dysfunction at rest.^15^ Moreover, an Asian study reported that, after stress echocardiography, only those patients with an LVEF of 55% at rest were confirmed to have systolic dysfunction.^11^ The average MELD score in previous studies on the Indian population was 12 in Anish et al.’s study and around 15 in Somani et al.’s study, whereas it was 18 in our study.^7^,^10^ The overt systolic dysfunction noted in our study may be explained as follows: Systolic dysfunction varies from hyperdynamic circulation resulting in increased LVEF in the initial stage to decreased cardiac output in the advanced stage of cirrhosis. Cardiac afterload progressively reduces as cirrhosis advances, but the proportional increase in heart rate does not happen because of the impaired chronotropic function to increased sympathetic activity. The decreased cardiac output predisposes to hepatorenal syndrome in decompensated cirrhosis.^1^,^2^ Nazar et al. observed significantly lower cardiac output in cirrhosis with ascites and in patients with markedly increased plasma renin than in those with normal or moderately increased renin, parallel to a previous study by Ruiz-del-Arbol et al.^16^,^17^ Furthermore, Krag et al. reported that systolic dysfunction in advanced cirrhosis was significantly associated with the hepatorenal syndrome and a poor survival rate.^18^ Systolic dysfunction was most frequent (*P*<0.05), and LVEF was significantly reduced (*P*=0.001) in the CTP-C stage of cirrhosis in our study. Moreover, patients with systolic dysfunction had significantly higher MELD scores (21.7±4.3 versus 17.1±4.0, *P*<0.001). However, the present study did not evaluate hepatorenal syndrome as it was not within the study protocol.

Diastolic dysfunction is a prominent and frequent feature of CCM. Diastolic dysfunction varies between 43% and 70% in cirrhotic patients, mostly of mild grade.^1^,^8^ Our study found DD in 45.9% of cases, of which 32.3% were of grade I. Diastolic dysfunction is usually detected by conventional echocardiography. But it has limitations because of a pseudo-normal pattern in advanced DD, and the E/A ratio is load-dependent. Tissue Doppler imaging can overcome these disadvantages. It is also included in the American Society of Echocardiography criteria for DD.^13^ Speckle tracking echocardiography and cardiac magnetic resonance are newer techniques but have not proved to be superior and are not widely used due to cost and availability.^19^ The postulated mechanism behind myocardial stiffness and impaired ventricular relaxation is mainly LVH in addition to fibrosis and sub-endocardial edema.^1^,^2^ A subsequent experimental study revealed the myofilament protein titin, which is responsible for the elasticity of cardiac muscles and which may play a role in the pathogenesis of DD.^3^ Cardiac hypertrophy was observed by Ruíz-del-Arbol et al. in 75% of patients with DD in cirrhotic patients.^20^ The present study observed LVH in 43.8% of patients, similar to the observation by Anish et al. in their study.^10^ Diastolic dysfunction was not related to cirrhosis etiology and severity in our study, consistent with many previous studies.^7^,^10^,^14^ Furthermore, a recent study by Lee et al. concluded that DD was not only found independent of the severity of cirrhosis, it was significantly associated with poor survival.^21^

Among the electrophysiologic abnormalities in cirrhosis, QT prolongation is the most common and is found in 30%–60% of cases.^1^ In the present study, it was found in 61.5% of patients, in agreement with other studies.^14^,^22^ The QT interval becomes prolonged primarily due to membrane ion channel dysfunction resulting in prolonged repolarization. Though many attributing factors like autonomic dysfunction and exposure to endotoxins, bile salts, and cytokines through porto-systemic shunts are postulated, the underlying pathophysiology has not yet been elucidated.^1^,^23^ In our study, QT interval prolongation was associated with cirrhosis severity (*P*<0.05), as established by many previous studies.^14^,^22^ Though the clinical significance is not clear, cirrhotic patients with prolonged QT intervals are at risk for fatal arrhythmia as they are frequently on diuretics, vasoactive drugs, and prophylactic fluoroquinolones. Beta-blockers can be used but have a deleterious effect in cirrhotic patients with refractory ascites.^1^,^23^ However, close monitoring for electrolyte imbalances and avoiding or reducing the dose of QT interval-prolonging drugs may be beneficial.

Cirrhotic cardiomyopathy is an independent predictor of overall survival in cirrhosis.^21^ With liver transplantation, cardiac events like arrhythmias, heart failure, and myocardial infarction have been reported in 25%–70% of cases.^1^,^24^ Moreover, DD is a risk factor for graft rejection and failure after liver transplantation.^25^ Further, a recent Indian study observed no survival benefit at three months after liver transplantation.^26^ Despite its significant morbidity and mortality, cirrhotic patients are not routinely screened for CCM. Our study suggests that CCM has a positive relationship with cirrhosis severity. An early approach with a routine cardiac evaluation with ECG and echocardiography, close monitoring, and targeted therapy toward underlying pathology leading to decompensation may prevent the complications of CCM.

A limitation of the present study is that it was a single-center study with a smaller sample size, raising the possibility of bias in patient selection. All contemporary methods were not used to assess the systolic and DD. In addition, participants were not subjected to stress echocardiography, and B-type natriuretic peptide was not estimated to detect subclinical cardiac dysfunction. Though the most common etiology of cirrhosis was alcohol in our study, none of the participants were actively drinking alcohol at the time of the study. Further, none of the cardiac changes were significantly different between non-alcoholic and alcoholic cirrhosis, indicating the results were apparently due to cirrhosis rather than the toxic effects of alcohol. However, longitudinal, multi-center studies with larger sample sizes are needed to validate the findings of our study.

## CONCLUSION

Cirrhotic cardiomyopathy frequently occurs in cirrhosis, irrespective of the etiology and demographic characteristics of patients. Moreover, it has a positive relationship with the severity of cirrhosis. Although DD is more frequent than systolic dysfunction, it is mainly of mild grade and not related to the severity of cirrhosis. In contrast, systolic function declines with the severity of cirrhosis, and overt systolic dysfunction can be present, particularly in the end-stage of liver cirrhosis.

## Abbreviations

CCM: cirrhotic cardiomyopathy
CTP: Child–Turcotte–Pugh
DD: diastolic dysfunction
DT: deceleration time
IVRT: isovolumetric relaxation time
LVEF: left ventricular ejection fraction
LVH: left ventricular hypertrophy
MELD: Model for End-stage Liver Disease
